# Exploring potential working mechanisms of accelerated HF-rTMS in refractory major depression with a focus on locus coeruleus connectivity

**DOI:** 10.1192/j.eurpsy.2024.1769

**Published:** 2024-10-17

**Authors:** Guo-Rong Wu, Chris Baeken

**Affiliations:** 1 Key Laboratory of Cognition and Personality, Faculty of Psychology, Southwest University, Chongqing, China; 2 Ghent Experimental Psychiatry (GHEP) Lab, Ghent University, Ghent, Belgium; 3 Faculty of Medicine and Health Sciences, Department of Head and Skin, Ghent University, Ghent, Belgium; 4 Vrije Universiteit Brussel (VUB), Department of Psychiatry, University Hospital (UZBrussel), Brussels, Belgium; 5 Department of Electrical Engineering, Eindhoven University of Technology Eindhoven, The Netherlands

**Keywords:** accelerated rTMS, locus coeruleus, major depressive disorder

## Abstract

**Background:**

This study investigates the effects of accelerated high-frequency repetitive transcranial magnetic stimulation (aHF-rTMS), applied to the left dorsolateral prefrontal cortex (DLPFC), on locus coeruleus (LC) functional connectivity in the treatment of refractory medication-resistant major depression (MRD).

**Methods:**

We studied 12 antidepressant-free refractory MRD patients, focusing on how aHF-rTMS affects the LC, a central component of the brain’s noradrenergic system and key to mood regulation and stress response.

**Results:**

A stronger decrease in LC functional connectivity following aHF-rTMS treatment resulted in better clinical improvement. We observed such LC functional connectivity decreases with several brain regions, including the superior frontal gyrus, precentral gyrus, middle occipital gyrus, and cerebellum. Moreover, our exploratory analyses hint at a possible role for E-field modeling in forecasting clinical outcomes. Additional analyses suggest potential genetic and dopaminergic factors influencing changes in LC functional connectivity in relation to clinical response.

**Conclusions:**

The findings of this study underscore the pivotal role of the LC in orchestrating higher cognitive functions through its extensive connections with the prefrontal cortices, facilitating decision-making, influencing attention, and addressing depressive rumination. Additionally, the observed enhancement in LC-(posterior) cerebellar connectivity not only highlights the cerebellum’s role in moderating clinical outcomes through noradrenergic system modulation but also suggests its potential as a predictive marker for aHF-rTMS efficacy. These results reveal new insights into the neural mechanisms of refractory depression and suggest therapeutic targets for enhancing noradrenergic activity, thereby addressing both cognitive and psychomotor symptoms associated with the disorder.

## Introduction

Accelerated repetitive transcranial magnetic stimulation (arTMS) represents a significant advancement in the non-invasive treatment of the major depressive disorder (MDD). This innovative approach has garnered attention for its potential to deliver therapeutic effects more rapidly than conventional rTMS protocols. The promise of arTMS lies in its ability to compress the treatment schedule without compromising efficacy, offering patients a faster route to symptom relief [1]. Recent research on arTMS also suggests that this type of application may be beneficial in severely depressed patients who do not respond to most of our available treatment options, including pharmacotherapy and psychotherapy, and even when electroconvulsive therapy (ECT) is not successful [2]. Although much attention is currently paid to predicting clinical outcomes, mostly based on specific symptoms or functional connectivity patterns, the neurobiological effects associated with clinical improvement lag behind, and especially in the refractory state.

Preclinical research suggests that rTMS shares underlying mechanisms with classical monoaminergic therapies, affecting inflammation, synaptic plasticity, neurogenesis, monoamine neurotransmitters, and the hypothalamic–pituitary–adrenal (HPA) system [3]. Human studies also show that classical antidepressant treatments primarily target monoamine systems, with secondary effects on other neurotransmitter systems and pathways, promoting neurogenesis and synaptic plasticity via upregulation of brain-derived neurotrophic factor (BDNF) [4]. Similarly, rTMS in MDD enhances synaptic plasticity and neurogenesis by activating BDNF and other pathways, directly modulating neuronal circuits and affecting multiple neurotransmitter systems, including glutamatergic and GABAergic systems, beyond monoamines [5, 6]. Some evidence from daily rTMS protocols suggests the involvement of the dopaminergic and serotonergic systems, but other neurotransmitter systems such as the noradrenergic system remain largely underexplored.

In pharmacotherapy, it is known that reduced norepinephrine (NE) release from the locus coeruleus (LC) can lead to characteristic depressive symptoms such as low energy, reduced motivation, and poor concentration. Furthermore, chronic stress is known to exacerbate depressive symptoms and disturbances in NE transmission from the LC [7]. Pharmaceutical treatments that inhibit NE reuptake are designed to increase synaptic NE levels and thereby alleviate depressive symptoms [8], and they have also been shown to induce structural and functional changes in the brain, particularly in regions connected to the LC [9].

To gain a deeper understanding of the potential noradrenergic mechanisms in the context of accelerated high-frequency rTMS (aHF-rTMS) treatment, we hypothesized that this would induce notable changes in LC functional connectivity, which in turn may alleviate symptoms of MRD. In addition, given the ambiguity surrounding the neurobiological underpinnings of these functional connectivity changes, we also explored potential influences from gene expression and neurotransmitter systems.

## Materials and methods

This study is part of a larger project exploring neurobiological markers of (a)HF-rTMS in MRD.

### Participants

The research received ethical approval from the ethics committee of UZBrussel, our university hospital. Twelve at least Stage III treatment-resistant antidepressant-free MDD patients, comprising 9 females and 3 males with an average age of 48.92 years (sd = 12.18 years) took part in the current study (see [10] for details). The mean duration of the current depressive episode was 7.88 years (sd = 7.17 years), confirming the refractory state. Three patients were ECT non-responders. Exclusion criteria included neurological diseases, a history of bipolar disorder, psychosis, substance dependency, or a recent suicide attempt within the last six months. Before undergoing aHF-rTMS treatment, none of the participants were on antidepressants, antipsychotics, or mood stabilizers for a minimum of two weeks. Changes in depression severity were measured using the 17-item Hamilton Depression Rating Scale (HDRS; [11]), which was administered by a psychiatrist not involved in the study.

### Study protocol

Stimulation was delivered using a Magstim rapid magnetic stimulator (Magstim Company Limited, Minnesota, USA) with a 70 mm diameter figure-of-eight coil placed over the left DLPFC under MRI guidance. The resting motor threshold (rMT) for the right abductor pollicis brevis muscle was established through electromyography and set at 110% of rMT for stimulation. The exact location for stimulation was pinpointed using three-dimensional magnetic resonance imaging (3D-MRI), identifying the mid-prefrontal gyrus’s central region as the target for the left DLPFC, and marked by MNI coordinates (−45, 30, 31) as noted in Wu and Baeken [12]. The coil was positioned tangentially to the skull, with its handle directed 45° antero-medially. To ensure patient blindness to the stimulation type, they were equipped with earplugs and blindfolds during the process. All patients underwent an accelerated HF-rTMS protocol at 20 Hz, spread over 4 days with five sessions each day. Each session included 40 trains lasting 1.9 seconds, interspersed with 12-second intervals, totaling 1560 pulses per session. The intersession interval was approximately 15–20 minutes. The patients first received active HF-rTMS in the first week, followed by a sham treatment the next week, or the other way around. As described in Baeken et al. [10], we only used the baseline and last fMRI scan at the end of the stimulation protocol.

### Brain imaging

The resting state BOLD fMRI data were preprocessed using fMRIPrep (version 1.5.0, [13]). Briefly, the T1-weighted (T1w) image underwent intensity nonuniformity correction and was segmented into cerebrospinal fluid (CSF), white matter (WM), and gray matter. Subsequently, the BOLD fMRI data were motion-corrected using mcflirt and co-registered with the T1w image. Following that, slice-time correction of the BOLD data was performed using 3dTshift, followed by resampling into the MNI152NLin2009cAsym standard volumetric space and spatial smoothing with a Gaussian kernel of 6-mm FWHM. Confounds including the six head-motion parameters and physiological noise estimated using the anatomical component correction method (aCompCor; top five principal components derived from CSF and WM masks extracted from the normalized data without spatial smoothing) were computed and regressed from the preprocessed BOLD fMRI data. Finally, temporal band-pass filtering between 0.01 Hz to 0.1 Hz was applied to the residual BOLD fMRI time series.

The seed-based functional connectivity maps of the LC were obtained by calculating Fisher-transformed correlation coefficients between average LC’s BOLD signals (extracted from the temporally filtered data without spatial smoothing) and signals in all other voxels (temporally filtered data that was also spatially smoothed), using the rsHRF toolbox [14]. The LC is located in the upper segment of the pons, adjacent to the base of the fourth ventricle, and can be identified utilizing the Harvard Ascending Arousal Network Atlas (https://www.nmr.mgh.harvard.edu/resources/aan-atlas, [15]).

The association between changes in LC seed-based functional connectivity (delta LC-FC = baseline LC FC – post stimulation LC-FC) and clinical outcomes (delta HDRS = HDRS post stimulation – HDRS baseline) was investigated using multiple regression analysis in SPM12, with age, sex, order (first real vs. sham aHF-rTMS or vice versa), and mean framewise displacement included as covariates. For multiple comparisons, statistical significance at *p* < 0.05 was adjusted using cluster-level family-wise error rate (FWE) correction.

Consistent with previous findings, we further examined whether there was an overlap between the multiple regression *t*-map (the association between alterations in LC functional connectivity and clinical outcomes) and the distribution of the electric field (E-field) as it may relate to clinical outcomes [12]. Electric field modeling was performed on MNI152 mesh using SimNIBS (version 3.2.6). The stimulation intensity, coil orientation (calculated for a Magstim 70-mm figure-of-eight coil), and coil-to-scalp distance were set to dI/dt = 1 A/ms, 45^o^ to midline, and 4 mm, respectively. The coil center was positioned directly above the left prefrontal stimulation site (DLPFC: MNI coordinates *x* = -45, *y* = 30, *z* = 31). Finally, we also explored whether the multiple regression *t*-map correlated with the distribution of neurotransmitter systems (as provided by the JuSpace toolbox [16]) and gene expressions (considering only 12 MDD-related genes from Allen Human Brain Atlas dataset: “ADRA2A,” “CHRM2,” “CNR1,” “CRH,” “CUX2,” “GAD2,” “HTR1A,” “HTR5A,” “MAOA,” “PDE1A,” “SST,” and “TAC1”). Spin permutation testing based on spherical rotations (5000 times) was used to assess statistical significance of spatial correlation analysis while accounting for spatial autocorrelation [17].

## Results

**Figure 1.**
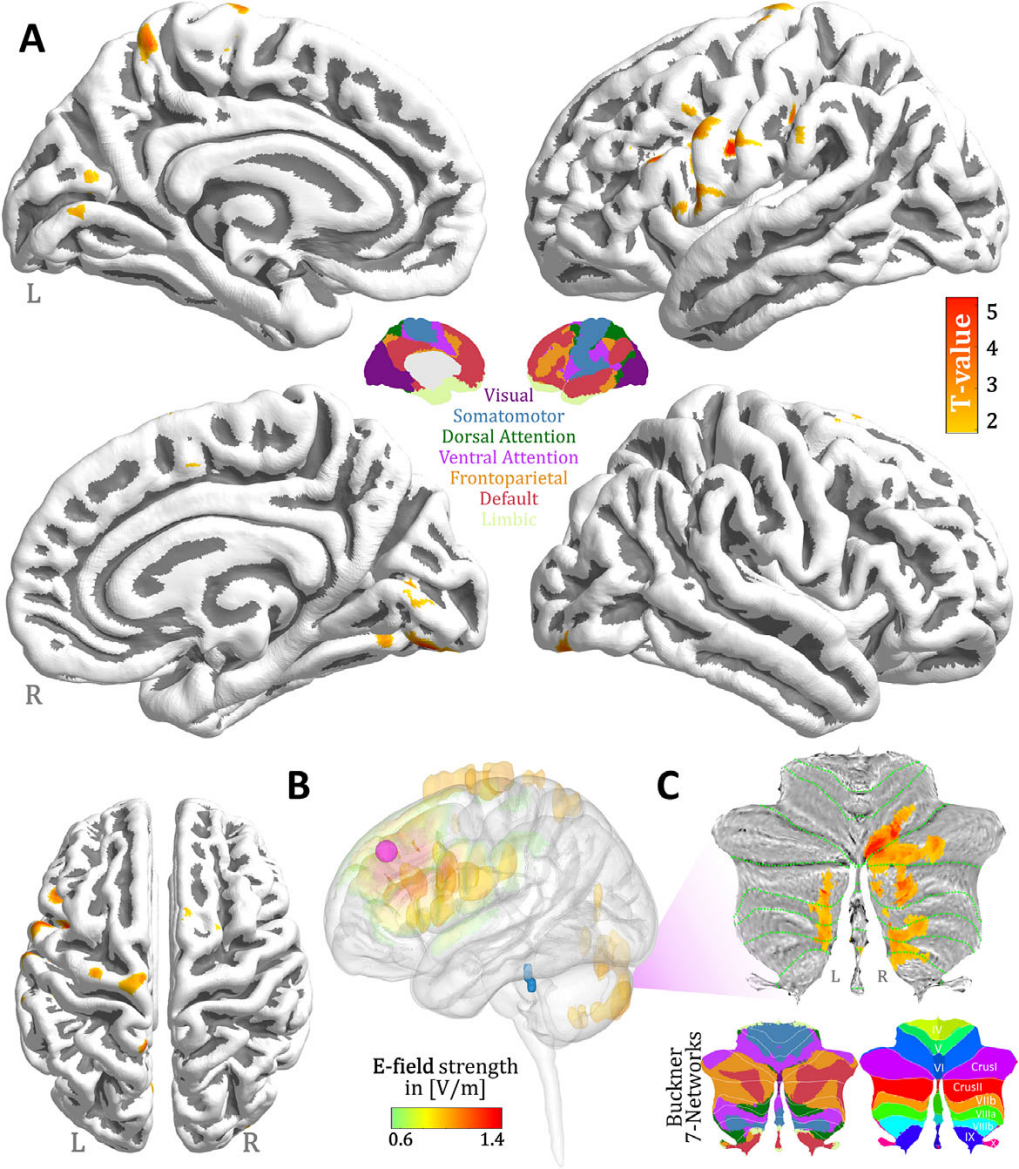
aHF-rTMS antidepressant treatment effects on Locus Coeruleus functional connectivity. (A) 3D brain renderings illustrate the regions resulting from the changes in LC functional connectivity with clinical improvement. Areas with significant positive correlation are marked in red. No significant negative correlation clusters were detected. For detailed statistical analysis of cluster size and location, we refer to Table 1. (B) Distribution of the electric field norm (displaying only the top 5th percentile value). The coil center (purple dot) was positioned directly above the left prefrontal stimulation site (DLPFC: MNI = 45, 30, 31). Visualization of the Locus Coeruleus in blue. (C) Top: Visualization of the positive correlation between the changes in LC functional connectivity within the cerebellar areas and delta HDRS projected to a cerebellar flatmap. Bottom: Buckner 7 network representations.

HDRS scores significantly declined post-treatment (*t*(11) = −3.9, *p* = 0.003). We found a significant positive correlation between clinical improvement following aHF-rTMS treatment and changes in LC functional connectivity with left-sided regions of the superior and precentral gyri, and the posterior cerebellum. This means that a stronger decrease in LC functional connectivity following aHF-rTMS treatment resulted in better clinical improvement. See also Table 1 and Figure 1.

**Table 1.** Locus Coeruleus functional connectivity changes related to clinical improvement following aHF-rTMS treatment in MDD

	Cluster size (#voxels)	Anatomical region	Hemisphere	*T*-value	Peak coordinates (x,y,z) (mm)
Positive	1073	Cerebellum Posterior lobe	Right	9.77	19, −89, −38
	987	Precentral Gyrus	Left	11.22	−54, −3, 26
	870	Superior Frontal Gyrus	Left	9.40	−21, −8, 76
Negative	No significant clusters emerged

We also found a significant spatial correlation between the E-field intensity and the LC functional connectivity change that corresponded to improved clinical outcomes (*r* = 0.19, Spin permutation test p_*FDR*_ = 0.03). This suggests that patients whose E-field and LC functional connectivity maps overlapped demonstrated better antidepressant outcomes than those without such an overlap. See also Figure 1B.

Lastly, a significant spatial correlation was observed in somatostatin (SST) interneurons, a major type of inhibitory neuron (*r* = 0.15, Spin permutation test p_*FDR*_ = 0.04); the CNR1 gene, which encodes for the CB1 cannabinoid receptor and is involved in modulating the activity of other neurotransmitter systems (*r* = 0.12, Spin permutation test p_*FDR*_ = 0.03); and the dopamine D1 antagonist SCH23390 (*r* = 0.14, Spin permutation test p_*FDR*_ = 0.02), suggesting a dopaminergic influence on the noradrenergic system. See also Supplementary Material.

## Discussion

This is the first study to examine locus coeruleus functional connectivity and accelerated HF-rTMS in a well-defined sample of medication-free refractory depressed patients.

First, the LC’s connection with the (left) superior frontal gyrus, a part of the dorsal attention network (DAN) and the frontoparietal control network (FPN), underscores its role in higher cognitive functions such as decision-making, attention, and rumination [18]. While the LC projects extensively throughout the brain, including subcortical regions [19], recent studies have also demonstrated reciprocal connections with the prefrontal cortices [20]. Although speculative, our results support the premise that stimulation of the (left) DLPFC may influence noradrenergic activity in the LC. Moreover, the LC’s relationship with the precentral gyrus, close to the stimulated area, within the somatomotor network reveals potential avenues for addressing psychomotor symptoms in MDD, often linked to disrupted LC noradrenergic function [21]. The LC-(posterior) cerebellar connectivity enhancement post-arTMS treatment, particularly with Crus I and Crus II areas within the FPN, underscores the cerebellum’s involvement in MRD’s clinical outcomes facilitated by the noradrenergic system modulation [22] but it also substantiates its role as a predictive marker for clinical outcomes for arTMS treatment in MDD [23].

Interestingly, patients who showed an overlap between their electric field (E-field) and the LC functional connectivity maps exhibited greater clinical improvements compared to those with lesser or no overlap. This suggests that evaluating the E-field distribution at the stimulation site might improve the prediction of patient responses to (aHF)rTMS therapy. Incorporating such imaging data can facilitate the creation of personalized treatment plans, potentially enhancing their effectiveness by customizing them to the individual neural characteristics of each patient [24].

Concerning gene expressions and neurotransmitter systems, the CNR1 gene is associated with changes in LC functional connectivity, affecting clinical outcomes in MDD, highlighting its significance in therapeutic interventions [25], potentially including aHF-rTMS. The interaction between SST interneurons and pyramidal neurons’ apical dendrites leads to synaptic and extrasynaptic inhibition, suggesting that aHF-rTMS could exert beneficial inhibitory effects on depression symptoms [26]. Supporting this, a previous aHF-rTMS study found that increased GABA levels in the stimulated area were linked to improved clinical outcomes in refractory depression [27]. Additionally, the D1-like dopamine receptor antagonist SCH23390 involvement suggests a complex interplay between the dopaminergic and noradrenergic systems, influencing cognitive and behavioral processes [28]. It is interesting to note that TMS long-term potentiation (LPT), a long-lasting enhancement of signaling between neurons, specifically requires the involvement of D1 receptors [29].

Both monoaminergic psychopharmacological treatments and rTMS offer significant therapeutic benefits for MDD by modulating neurotransmitter levels and enhancing neuroplasticity. However, the multifaceted approach of (a)rTMS may offer additional benefits that may explain its efficacy in patients who do not respond to traditional monoaminergic treatments. This broader effect profile, although speculative, may contribute to its efficacy in treatment-resistant cases.

Of course, our study has obvious limitations: (1) The study involved only 12 patients, and all were of the refractory type, so it remains to be explored if our finding can be extrapolated to less resistant MDD patients. (2) Although the Harvard Ascending Arousal Network Atlas clearly defines the LC, because it is a small region, individualized LC masks may be preferred [30]. 3) While our investigation explored genetic factors and various neurotransmitter systems that may influence the activity of the LC noradrenergic system, it is important to note that these interpretations regarding the neurobiological mechanisms underlying aHF-rTMS remain speculative.

This aHF-rTMS study underscores the significance of the LC functional connectivity with brain regions crucial for cognitive and emotional regulation. Our findings collectively suggest a multifaceted impact of aHF-rTMS treatment on MRD, highlighting the importance of LC connectivity across various brain networks in mediating clinical improvements. They also emphasize the importance of personalized treatment plans based on electric field distribution and highlight the role of genetic and neurotransmitter systems in therapeutic outcomes. Despite promising results, limitations such as small sample size and speculative neurobiological mechanisms call for cautious interpretation and further research.

## Supporting information

Wu and Baeken supplementary material Wu and Baeken supplementary material

## Supplementary material

The supplementary material for this article can be found at http://doi.org/10.1192/j.eurpsy.2024.1769.
