# Supplementary material for: Exploring potential working mechanisms of accelerated HF-rTMS in refractory major depression with a focus on locus coeruleus connectivity
Source: Eur Psychiatry. 2024 Oct 17;67(1):e70. doi: 10.1192/j.eurpsy.2024.1769 (PMC11730058; doi:10.1192/j.eurpsy.2024.1769)
Supplement: Wu and Baeken supplementary material [file S0924933824017693sup001.docx]

**Supplementary Material**

| genes | $r$ | $p$-value (uncorrected) |
| --- | --- | --- |
| ADRA2A | 0.031410985 | 0.469306139 |
| CHRM2 | 0.029524546 | 0.422315537 |
| CNR1 | 0.118925944 | **0.00219956 |
| CRH | 0.063823402 | 0.093381324 |
| CUX2 | 0.000116716 | 0.99780044 |
| GAD2 | 0.041099705 | 0.285342931 |
| HTR1A | 0.066349268 | 0.051789642 |
| HTR5A | 0.077173553 | 0.071185763 |
| MAOA | -0.069314644 | 0.163367327 |
| PDE1A | 0.039605018 | 0.323335333 |
| SST | 0.146992296 | **0.00679864 |
| TAC1 | 0.042117663 | 0.304939012 |

**Table S1.** The association between gene expression distribution and the statistical map depicting the correlation of alterations in LC functional connectivity with clinical outcomes. ** $p$ <0.01.

| PET receptor/neurotransmitter/activity maps | $r$ | $p$-value (uncorrected) | Source Templates |
| --- | --- | --- | --- |
| 5HT1a (cumi) | 0.047367208 | 0.082783443 | 5HT1a_cumi_hc8_beliveau |
| 5HT1a (WAY) | 0.080682509 | *0.023395321 | 5HT1a_WAY_HC36 |
| 5HT1b (az) | 0.015623954 | 0.579484103 | 5HT1b_az_hc36_beliveau |
| 5HT1b (P943) | 0.016538652 | 0.641071786 | 5HT1b_P943_HC22 |
| 5HT2a (ALT) | 0.055052698 | 0.088782244 | 5HT2a_ALT_HC19 |
| 5HT2a (cimbi) | 0.04100189 | 0.132973405 | 5HT2a_cimbi_hc29_beliveau |
| 5HT4 (sb20) | 0.045648362 | 0.083183363 | 5HT4_sb20_hc59_beliveau |
| CB1 (FMPEPd2) | 0.080069304 | *0.020395921 | CB1_FMPEPd2_hc22_laurikainen |
| CBF (ASL) | 0.084757373 | *0.014197161 | CBF_ASL_MRI |
| D1 (SCH23390) | 0.144061252 | ***0.00079984 | D1_SCH23390_c11 |
| D2 (fallypride) | -0.040132873 | 0.292341532 | D2_fallypride_hc49_jaworska |
| D2 (RACLOPRIDE) | -0.045975335 | 0.106378724 | D2_RACLOPRIDE_c11 |
| DAT (DATSPECT) | -0.035763737 | 0.326334733 | DAT_DATSPECT |
| FDOPA (f18) | 0.056100518 | 0.073785243 | FDOPA_f18 |
| GABAa (FLUMAZENIL) | 0.069642022 | 0.071985603 | GABAa_FLUMAZENIL_c11 |
| GABAa (flumazenil) | -0.019784924 | 0.48970206 | GABAa_flumazenil_hc16_norgaard |
| KappaOp (LY2795050) | 0.013076677 | 0.684463107 | KappaOp_LY2795050_hc10_ShokriKojori |
| mGluR5 (abp) | 0.078526437 | *0.037792442 | mGluR5_abp_hc22_rosaneto |
| mGluR5 (abp) | 0.079778358 | *0.031793641 | mGluR5_abp_hc28_dubois |
| mGluR5 (abp) | 0.084672965 | *0.025994801 | mGluR5_abp_hc73_smart |
| MU (CARFENTANIL) | 0.093284428 | **0.00919816 | MU_CARFENTANIL_c11 |
| MU (carfentanil) | 0.07361906 | 0.050789842 | MU_carfentanil_hc39_turtonen |
| NAT (MRB) | 0.001189831 | 0.968606279 | NAT_MRB_c11 |
| NMDA (ge179) | 0.073026888 | *0.038192362 | NMDA_ge179_29hc_galovic2021 |
| SERT (dasb) | 0.018071169 | 0.547090582 | SERT_dasb_hc100_beliveau |
| SERT (DASB) | 0.001959552 | 0.950209958 | SERT_DASB_HC30 |
| SERT (MADAM) | 0.004981164 | 0.881223755 | SERT_MADAM_c11 |
| VAChT (feobv) | 0.014978179 | 0.677464507 | VAChT_feobv_hc18_aghourian |
| VAChT (feobv) | -0.031722113 | 0.383323335 | VAChT_feobv_hc4_tuominen |
| VAChT (feobv) | 0.009252691 | 0.778044391 | VAChT_feobv_hc5_bedard |

**Table S2**. The association between neurotransmitter distribution and the statistical map depicting the correlation of alterations in LC functional connectivity with clinical outcomes. .* $p$<0.05, ** $p$ <0.01, *** $p$ <0.001.


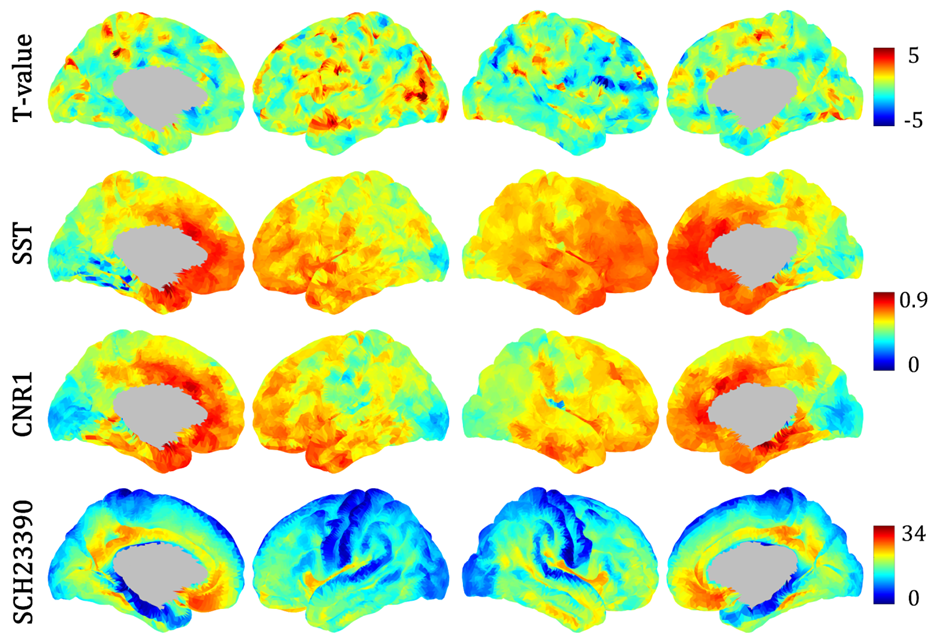


**Figure S1.** Spatial distribution of T-values (demonstrating the correlation between changes in LC functional connectivity and clinical improvement), gene expression, and neurotransmitter maps.
